# GOA-optimized deep learning for soybean yield estimation using multi-source remote sensing data

**DOI:** 10.1038/s41598-024-57278-6

**Published:** 2024-03-26

**Authors:** Jian Lu, Hongkun Fu, Xuhui Tang, Zhao Liu, Jujian Huang, Wenlong Zou, Hui Chen, Yue Sun, Xiangyu Ning, Jian Li

**Affiliations:** 1 Institute of Smart Agriculture, Jilin Agricultural University, Changchun, 130118 People’s Republic of China; 2 College of Agriculture, Jilin Agricultural University, Changchun, 130118 People’s Republic of China; 3 College of Information Technology, Jilin Agricultural University, Changchun, 130118 People’s Republic of China; 4 Northeast Institute of Geography and Agroecology, Chinese Academy of Sciences, Changchun, 130102 People’s Republic of China; 5 College of Surveying and Exploration, Jilin Jianzhu University, Changchun, 130119 People’s Republic of China

**Keywords:** GOA, Deep learning framework, Multi-source remote sensing data, Soybean yield estimation, Photosynthesis-related parameters, Computer science, Agroecology

## Abstract

Accurately estimating large-area crop yields, especially for soybeans, is essential for addressing global food security challenges. This study introduces a deep learning framework that focuses on precise county-level soybean yield estimation in the United States. It utilizes a wide range of multi-variable remote sensing data. The model used in this study is a state-of-the-art CNN-BiGRU model, which is enhanced by the GOA and a novel attention mechanism (GCBA). This model excels in handling intricate time series and diverse remote sensing datasets. Compared to five leading machine learning and deep learning models, our GCBA model demonstrates superior performance, particularly in the 2019 and 2020 evaluations, achieving remarkable R^2^, RMSE, MAE and MAPE values. This sets a new benchmark in yield estimation accuracy. Importantly, the study highlights the significance of integrating multi-source remote sensing data. It reveals that synthesizing information from various sensors and incorporating photosynthesis-related parameters significantly enhances yield estimation precision. These advancements not only provide transformative insights for precision agricultural management but also establish a solid scientific foundation for informed decision-making in global agricultural production and food security.

## Introduction

In global agricultural and economic contexts, soybeans serve as a crucial source for food and feed, and additionally, as a fundamental raw material for diverse industrial products. The stability of soybean supplies exerts considerable influence on global markets and food security concerns^1,2^. In light of ongoing climate change and increasing global population, the assurance of a stable supply of key crops, including soybeans, has garnered international attention^3,4^. Consequently, the accurate estimation of soybean yields across extensive areas holds substantial relevance for ensuring food security and promoting sustainable agricultural practices.

Traditional yield estimation methods predominantly rely on ground data collection, including field surveys and sampling. However, implementing these methods across extensive areas proves challenging, being both time-intensive and laborious^5,6^. Furthermore, geographical and climatic constraints often limit data collection coverage and frequency, potentially diminishing the timeliness and accuracy of the information gathered. Yield estimations also utilize process-oriented crop simulation models and statistical-based models. The former requires detailed analysis of factors influencing crop growth such as soil quality, climatic conditions, and cultivation management^7^. While these models offer in-depth insights into crop growth processes, their effectiveness is often constrained by the need for extensive field data and limited adaptability in complex environmental scenarios. Conversely, statistical models use statistical correlations between crop yields and variables like weather conditions and soil types for estimation^8^. Though sometimes accurate, their major drawback is the inability to fully encapsulate the intricate dynamic interactions between crop growth and environmental factors^9^. Given these complexities, there is a pressing need for more sophisticated and holistic approaches to mitigate uncertainties in soybean production, thereby enhancing the effectiveness of estimation and the ability to respond to potential yield fluctuations.

The incorporation of machine learning and deep learning techniques in crop yield estimation has markedly outperformed traditional methodologies^10,11^. These advanced technologies are adept at analyzing a vast and varied range of data, offering more complete and accurate insights^12^. They adeptly discern subtle correlations between crop growth and environmental variables through sophisticated algorithmic models. This enables a nuanced interpretation of complex non-linear relationships and effective yield estimation under diverse environmental scenarios^13,14^. For instance, Zhu et al. utilized two machine learning models—Random Forest Regression (RFR) and Support Vector Regression (SVR)—alongside four deep learning models, including Deep Neural Network (DNN), Convolutional Neural Network (CNN), Long Short-Term Memory (LSTM), and Deep Learning Adaptive Crop Model (DACM), for soybean yield estimation in the United States^15^. Their results affirmed the efficacy of these models in large-scale crop yield forecasting. Additionally, the study by Wang et al. further advances this domain^16^. They formulated a hybrid CNN and Gated Recurrent Unit (GRU) model specifically for winter wheat yield estimation and benchmarked it against the traditional GRU model. Their findings revealed that the CNN-GRU model not only outperformed the GRU in predictive accuracy but also demonstrated enhanced stability and generalizability across various crop years. These investigations highlight the significant potential of machine learning and deep learning in refining yield estimation accuracy, also indicating substantial opportunities for future technological advancements and optimizations.

In the realm of machine learning and deep learning applications, parameter optimization emerges as a critical factor in augmenting model performance^17^. This stage, characterized by the utilization of diverse optimization algorithms, holds significant importance in crop yield estimation. Recent research indicates that precision in tuning parameters, such as the learning rate, network layers, and neuron count, has led to enhanced estimation accuracy^18,19^. This precision tuning aids models in more effectively decoding the intricate interplay between crop growth and environmental influences and in adapting to yield estimations across various environmental scenarios. For instance, Zhang et al. employed a CatBoost regression model, optimized through Bayesian optimization, to predict winter wheat yield, demonstrating superior accuracy over other data-driven methods^20^. Similarly, Ali et al. enhanced wheat yield estimation accuracy by integrating an Online Sequential Extreme Learning Machine model with ant colony optimization algorithms^21^. Beyond Bayesian and ant colony optimization algorithms, other techniques like the Grasshopper Optimization Algorithm (GOA) have exhibited exceptional efficacy in model optimization. GOA, inspired by grasshoppers' swarming behavior, excels in finding both global and local optima, thereby improving efficiency and precision in parameter tuning^22,23^. Its capabilities are particularly advantageous in multi-dimensional data estimations, offering accelerated optimization processes and improved adaptability to varying environmental and data conditions^24^. Compared to other algorithms, GOA ensures more robust model performance in diverse settings, a benefit for researchers and practitioners with limited time and resources^25,26^. Therefore, we propose employing GOA in yield estimation to capitalize on its strengths in handling multi-dimensional data, aiming to enhance the model's efficiency and accuracy.

Crop yield estimation is inherently complex, involving an array of variables and multi-level factors^27^. Presently, numerous studies have ventured into using multi-source remote sensing data for yield estimation. For instance, Cheng et al. integrated indicators like Gross Primary Productivity (Gpp), Evapotranspiration (Et), Surface Temperature (Ts), Leaf Area Index (Lai), and soil properties with machine learning algorithms to estimate regional corn yield variations in China^28^. Li et al. employed vegetation indices, alongside soil and climate data, for wheat yield estimation in China, showing that remote sensing vegetation indices significantly enhance model accuracy^29^. Employing multi-source data has notably improved estimation accuracy, particularly in assessing large-scale crop growth conditions and environmental impacts^30^. However, despite the relevance of factors like vegetation indices, climatic conditions, and soil characteristics, their predictive capacity is not fully optimized. Recognizing that photosynthesis is the central process in plant growth and development, and a direct determinant of crop yield and quality, incorporating photosynthesis parameters into yield estimation models could markedly improve their precision and effectiveness^31^. Numerous researchers are now leveraging light and related parameters for this purpose. For example, some studies have explored the potential of Solar-Induced chlorophyll Fluorescence (SIF) in crop yield estimation, as SIF directly reflects plant photosynthetic activity and provides insights into plant growth status^32,33^. Additionally, GPP, as an essential measure of crop photosynthesis and carbon fixation, has been utilized for predicting crop yields, offering vital information about crop growth conditions^34,35^. Moreover, various photosynthesis-related parameters such as LAI, Fraction of Photosynthetically Active Radiation (FPAR), SIF, GPP, and Net Photosynthesis (PsnNet) are often underexploited, despite their direct link to crop light use efficiency and carbon fixation capacity^36–38^. While these parameters hold significant potential for precise yield estimation, their comprehensive utilization in current research remains limited.

This research employs multi-source remote sensing data—encompassing surface reflectance data, vegetation indices, environmental data, and photosynthesis-related parameters—to estimate soybean yields at the county level across the United States. The primary aim is to provide more accurate and generalizable soybean yield estimates across various counties. This endeavor not only bolsters the precision of yield estimates but also serves as a benchmark for crop yield estimation in other geographic areas. The specific objectives of this paper are fourfold: (1) To integrate multi-source remote sensing data with the GOA and the CNN-BiGRU-Attention model for assessing soybean yields at the county level in the United States. (2) To evaluate the performance of this model relative to other existing models. (3) To analyze the influence of different data sources on the accuracy of crop yield estimation. (4) To examine the role of photosynthesis-related parameters in soybean yield estimation.

## Material and methods

### Study area

**Figure 1:**
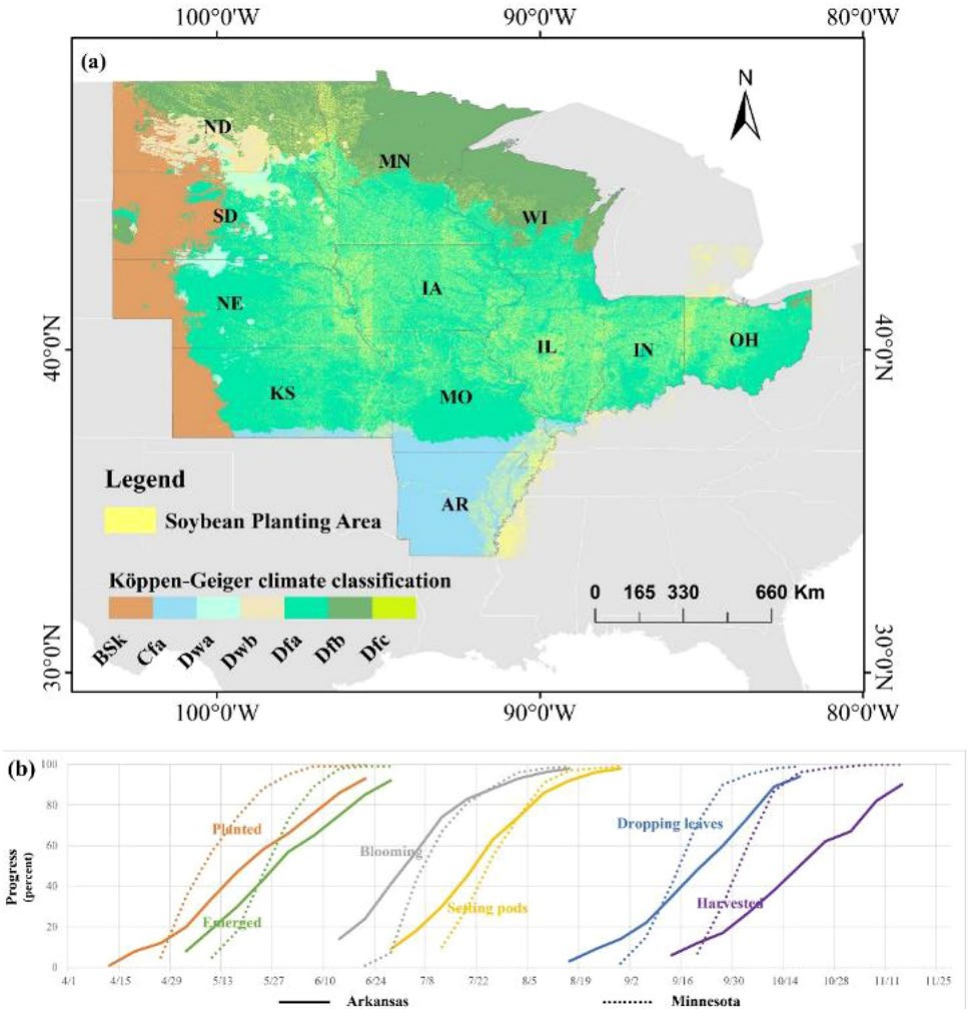
Overview of the study area. (**a**) Soybean distribution and climate distribution in 2020. The yellow cloud-shaped distribution represents the planting distribution of soybeans in the United States. The colored stripes indicate different climate types in the study area according to the Köppen-Geiger climate classification system. This system divides climates into several main types, including: BSk (Arid, steppe, cold), Cfa (Temperate, no dry season, hot summer), Dwa (Cold, dry winter, hot summer), Dwb (Cold, dry winter, warm summer), Dfa (Cold, no dry season, hot summer), Dfb (Cold, no dry season, warm summer), and Dfc (Cold, no dry season, cold summer). (**b**) Soybean growth progress in 2020 for the states of Arkansas and Minnesota. These data are sourced from the Crop Progress Reports of the United States National Agricultural Statistics Service (https://usda.library.cornell.edu/).

This study concentrates on twelve principal soybean-producing regions in the United States, strategically situated in the Central and Upper Midwestern parts of the country. These regions include Arkansas (AR), Illinois (IL), Indiana (IN), Iowa (IA), Kansas (KS), Minnesota (MN), Missouri (MO), Nebraska (NE), North Dakota (ND), Ohio (OH), South Dakota (SD), and Wisconsin (WI) as shown in Fig. 1a. These states exhibit notable climate variations, ranging from the humid subtropical climate of Arkansas to the temperate continental climate of Wisconsin. Such climatic diversity significantly influences the growth cycle and cultivation practices of soybeans. For instance, there are discernible variances in phenological stages and planting schedules between southern states like Arkansas and northern states like Minnesota. These differences not only affect key growth phases of soybeans, such as germination, flowering, podding, and leaf drop, but also have implications on the timing of harvest as depicted in Fig. 1b.

### Dataset and preprocessing

In this research, we gathered data spanning from May to August for each year between 2008 and 2020 to predict county-level soybean yields. This period was specifically chosen to account for the climatic variations among states, which influence the soybean planting and harvesting schedules. This timeframe is critical as it encapsulates the key stages of the soybean growth cycle, providing essential insights for yield estimation. Additionally, in terms of field crop management, particularly in extensive agricultural areas, predicting end-of-season yield well in advance of harvest—ideally 1–2 months prior—is a significant goal, as indicated by prior studies^39,40^. Our initial step involved standardizing the spatial and temporal resolution of the collected data—reflectance data, vegetation indices, environmental data, and photosynthesis-related data—to a uniform scale of 500 m and monthly intervals, respectively. Following this, we utilized a soybean planting area mask to annualize the data, subsequently aggregating it at the county level. All data preprocessing was conducted using the Google Earth Engine (GEE) platform ( https://earthengine.google.com/), which is renowned for its extensive repository of complimentary resources (including satellite imagery, climate data, topographic information, and atmospheric data) and its robust computational capabilities. The GEE platform was instrumental in this study, enabling efficient processing and analysis of the voluminous geospatial data, thus ensuring the research's accuracy and efficiency. The specifics of the data utilized in this study are detailed in Table 1.

**Table 1:** Sources of data.

Category	Variables	Temporal resolution	Spatial resolution	Time coverage	Data source
Soybean yield and planting area	County yield	Year	County	2008–2020 May–August	https://www.nass.usda.gov/
	Planting area (The Cropland Data Layer(CDL))	Year	30 m	2008–2020 May–August	https://www.nass.usda.gov/
Surface reflectance and vegetation indices	Surface reflectance(Sur_Refl_b01 to Sur_Refl_b07)	Daily	500 m	2008–2020 May–August	MOD09GA Version 6.1 product
	Vegetation index(NDVI,EVI)	16-day	500 m	2008–2020 May–August	MOD13A1 Version 6.1 product
Environmental data	LST (LST_Day_1km, LST_Night_1km)	Daily	1 km	2008–2020 May–August	MOD11A1 Version 6.1 product
	Climate data (Pdsi,Pr,Soil,Vap,Vpd)	Monthly	4 km	2008–2020 May–August	TerraClimate datasets
Photosynthetic related parameters	Sif	8-day	0.05°	2008–2020 May–August	RTSIF Chen Xingan^41^
	Fpar	4-day	500 m	2008–2020 May–August	MCD15A3H Version 6.1 product
	Lai	4-day	500 m	2008–2020 May–August	MCD15A3H Version 6.1 product
	Gpp	8-day	500 m	2008–2020 May–August	MOD17A2H Version 6.1 product
	PsnNet	8-day	500 m	2008–2020 May–August	MOD17A2H Version 6.1 product

#### Soybean yield and planting area

The soybean yield data for each county in the United States, spanning from 2008 to 2017, was acquired from the National Agricultural Statistics Service (NASS) of the United States Department of Agriculture (USDA) (https://www.nass.usda.gov/). The yields were measured in bushels per acre, where 1 bushel per acre is approximately equivalent to 67.25 kg per hectare. In certain states, counties with limited survey results were amalgamated into a statistical category named 'OTHER (combined) Counties.' To maintain the integrity and accuracy of our analysis, these aggregated data records were excluded from this study. This decision was made to prevent any potential misinterpretation or misleading conclusions that could arise from the generalized nature of this combined data.

In our research, The Cropland Data Layer (CDL) was employed as the primary source of crop classification data. CDL, an annual crop classification dataset, leverages satellite remote sensing technology to provide detailed crop type information. It offers a high resolution of 30 m, enabling precise identification of individual fields, which is particularly advantageous for county-level or more granular analyses. The dataset encompasses a wide range of crop types, including key crops such as soybeans, corn, and wheat, among others. This diversity furnishes a comprehensive resource for examining specific crop planting patterns.

For the purposes of this study, the CDL data was pivotal in pinpointing soybean planting areas. This strategic use was intended to reduce the potential impact of other crops and natural vegetation on our research findings. Additionally, we performed an initial quality assessment of both the yield data and the CDL data to mitigate data absence issues. This process involved two key checks: (1) Identifying and addressing instances of completely missing or discontinuous yield data; and (2) Examining and rectifying data points that deviated significantly from the average, specifically those falling outside the range of the average plus or minus two standard deviations for the years 2008 to 2020^42–44^.

#### Surface reflectance and vegetation indices

Surface Reflectance data for this study were sourced from the MOD09GA Version 6.1 product. This dataset is pivotal in representing the Earth's surface's capacity to reflect solar radiation, a vital aspect for analyzing surface characteristics, vegetation coverage, and environmental changes. It boasts a spatial resolution of 500 m. For our analysis, we selected seven distinct wavelength bands (Sur_Refl_b01 to Sur_Refl_b07), spanning from May to August for the years 2008 to 2020.

In addition, we incorporated two vegetation indices into our study—NDVI (Normalized Difference Vegetation Index) and EVI (Enhanced Vegetation Index). These indices were obtained from the MOD13A1 Version 6.1 product, which provides a 16-day temporal resolution at 500 m spatial resolution. NDVI and EVI are instrumental in evaluating and monitoring vegetation health and growth status^45^. NDVI is derived from the ratio of red and near-infrared light reflectance. EVI, building on NDVI, includes adjustments for atmospheric disturbances, land background signal, and vegetation structure. These indices are essential for comprehending ecosystem dynamics, tracking agricultural production trends, and understanding the impacts of climate change^46–48^.

#### Environmental data

In our study, we incorporated environmental data from two primary satellite sources: Land Surface Temperature (LST) and Climate data. The LST data were procured from the MOD11A1 Version 6.1 product. This dataset offers a spatial resolution of 1 km and includes daily measurements of land surface temperature during both day (LST_Day_1km) and night (LST_Night_1km). LST plays a critical role in comprehending surface energy balance, ecosystem health, and plant physiology, particularly within agricultural settings^49,50^. It is valuable for understanding surface heating trends, microclimate conditions, and in evaluating crop stress due to temperature fluctuations.

The Climate data were derived from the TerraClimate dataset. This comprehensive dataset provides monthly climate information, encompassing parameters such as the Palmer Drought Severity Index (Pdsi), precipitation (Pr), soil moisture (Soil), vapor pressure (Vap), and vapor pressure deficit (Vpd). We selected this dataset for the period from 2008 to 2020, with a spatial resolution of ~4-km (1/24th degree), and focused our analysis on the critical growing months from May to August. These datasets are crucial for understanding long-term climate trends, assessing drought conditions, and analyzing factors like water availability and moisture stress. These elements are fundamental in agricultural productivity studies and ecological research^51^.

#### Photosynthetic-related parameters

In this research, we utilized the Reconstructed TROPOMI SIF (RTSIF) product, a comprehensive global SIF dataset, for the period from May to August annually, spanning 2008 to 2020. This dataset, updated every 8 days at a spatial resolution of 0.05°, is generated using the XGBoost machine learning model. Inputs for the RTSIF include MODIS surface reflectance data, land surface temperature, land cover products, CERES reanalysis data, and C3/C4 vegetation cover data. The objective of RTSIF is to reconstruct TROPOMI SIF under clear sky conditions for the years 2001 to 2020. To ensure its reliability, RTSIF has undergone comparative analysis with tower-based SIF observations and other satellite-derived SIF datasets (GOME-2 SIF and OCO-2 SIF), demonstrating its high accuracy^52^. Additionally, we selected the MCD15A3H product for Fraction of Photosynthetically Active Radiation (Fpar) and Leaf Area Index (Lai), both of which have a spatial resolution of 500 m. The Fpar data is essential for estimating the amount of solar radiation absorbed by the photosynthetic canopy^53^, while Lai data offers valuable insights into leaf biomass density, crucial for understanding plant growth, canopy structure, and overall ecosystem productivity^54^. We also included Gpp and PsnNet data from the MOD17A2H product, which provides data at an 8-day frequency, consistent with the spatial and temporal scope of our study. These parameters are critical for quantifying carbon fixation during the ecosystem photosynthesis process, thus offering key insights into ecosystem productivity and carbon dynamics^55,56^.

### Methods

**Figure 2:**
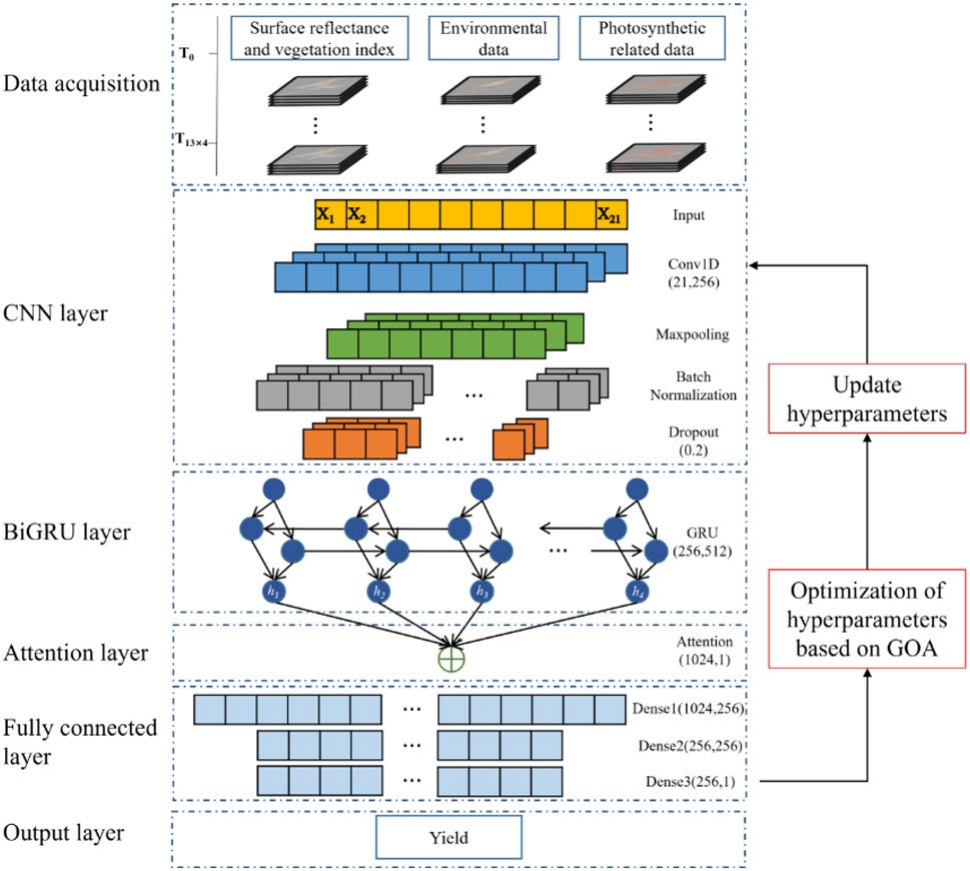
Network structure of the GCBA model for soybean yield estimation.

The primary goal of this study is to facilitate early soybean yield estimations during their critical growth period (May to August) by synergizing multi-source data with advanced deep learning techniques. For this purpose, we have developed a novel deep learning framework that integrates the GOA with a CNN and a Bidirectional Gated Recurrent Unit (BiGRU), further augmented by an Attention mechanism to boost the model's predictive accuracy. The architecture of this model is depicted in Fig. 2. To evaluate the effectiveness and superiority of our proposed GOA-CNN-BiGRU-Attention (GCBA) framework, we conducted comparative analyses using an array of machine learning and deep learning models as benchmarks. These comparative models include two machine learning methods, SVR and RFR, along with three deep learning networks, CNN, GRU, and a combined CNN + GRU approach. They were utilized to validate the enhanced performance capabilities of our GCBA framework.

#### GOA-CNN-BiGRU-attention framework

The innovative GCBA framework proposed in this study represents a significant advancement in the early estimation of soybean yields over extensive areas. This framework synergizes multi-source remote sensing data with cutting-edge deep learning technologies to adeptly capture the intricate spatial and temporal dynamics that influence soybean yield. Within the GCBA framework, the multi-source remote sensing data provide an all-encompassing perspective of the soybean growth environment. This encompasses a range of information, including surface reflectance, vegetation indices, climate data, and light-related parameters. These diverse datasets are then input into our specially designed deep learning model, which consists of several layers tailored to process this information effectively. The model employs a CNN layer for extracting pivotal features from the remote sensing data. The BiGRU layer is adept at analyzing time series data, capturing temporal patterns crucial for yield estimation. Furthermore, an attention mechanism is integrated within the model to focus on the most significant aspects of the data, enhancing the overall predictive accuracy. Concurrently, the model's hyperparameters are finely tuned using the GOA. GOA, inspired by the foraging behavior of grasshoppers, effectively amalgamates global and local search strategies to determine optimal network parameters. This methodology strikes a balance between exploration and exploitation, thereby preventing overfitting to the training data and enhancing the model's generalization capabilities on new, unseen data.

In the deep learning framework employed in this study, the CNN layer is pivotal, tasked with processing and interpreting the multi-source remote sensing data. The input dimensions of the CNN layer are configured to align with the number of features in the remote sensing data (21). Utilizing 256 output channels, the CNN layer efficiently extracts a diverse array of spatial features from the input data. Each channel incorporates a size 3 convolutional kernel, adeptly capturing local spatial relationships within the data. Following the convolutional operations, a 2 × 2 pooling kernel is applied for downsampling. This step is crucial as it reduces the feature dimensions, thereby enhancing the computational efficiency of the model. The resulting pooled feature maps are then processed through a Batch Normalization (BN) layer. The BN layer plays a key role in stabilizing the learning process by normalizing and scaling the activation values. This contributes to faster training speeds and aids in preventing overfitting. Subsequently, a Rectified Linear Unit (ReLU) activation function is introduced. ReLU, by setting all negative values to zero, induces non-linearity in the network. This feature enables the network to learn and represent complex patterns more effectively. The inclusion of ReLU also introduces sparsity among neurons, thereby increasing the model's efficiency and efficacy. These sequential operations not only bolster the feature extraction process but also ensure the generation of high-quality feature representations. These are then relayed to the subsequent BiGRU layer, forming a robust foundation for the model's overall learning and predictive capabilities. Moreover, to further enhance the model's generalization ability and mitigate the risk of overfitting, a Dropout layer is integrated with a rate of 0.2.

In our deep learning framework, the BiGRU layer assumes a critical role. This layer is composed of bi-directional GRU units, one processing the forward (past to future) sequence and the other handling the backward (future to past) sequence. Such a configuration enables the network to capture dependencies in the time-series data from both directions, offering a more nuanced and comprehensive contextual understanding. Within each GRU unit, the internal state is dynamically adjusted to prioritize relevant information while filtering out the extraneous, a process managed by internal update and reset gates. Our BiGRU layer incorporates 512 hidden units, distributed evenly across three GRU layers, each further refining the processing and transference of information, thereby enhancing the deep learning network's training. The output from the BiGRU layer is an abstracted feature representation that integrates information across multiple time steps, laying a solid foundation for accurate yield estimation. Following the BiGRU, an attention layer is employed. This layer calculates the significance weights for each element in the input sequence relative to the output, highlighting the most critical time points for crop yield estimation. It assigns a weight to each time step, which is then applied to the BiGRU output to create a weighted feature representation. This approach allows the model to concentrate more on pivotal time steps, enhancing the overall estimation accuracy. Subsequent to the attention layer is a fully connected layer, designed to analyze and learn from the attention-weighted features. This layer functions by receiving activations from its preceding layer and delivering outputs to the subsequent layer. Each unit in the fully connected layer is interconnected with all activations from the prior layer, facilitating the learning of global patterns from the input features. Our model includes three layers in the fully connected section. The first layer, with 1024 input units and 256 output units, is tasked with extracting higher-level abstractions from the attention-processed features. The second layer, consisting of 256 input and output units, further refines the feature representation to capture intricate relationships within the data. The final layer, the output layer, with 256 input units and a single output unit, is responsible for generating the ultimate county-level soybean yield estimations.

Prior to their introduction into the model, all datasets were normalized to a range between 0 and 1. This normalization step is crucial for ensuring consistent data scales and improving the model's learning efficiency. The GCBA model, formulated on the Pytorch framework, underwent an extensive training process spanning 200 epochs. For the loss function, we opted for HuberLoss, renowned for its robustness, particularly with regression tasks. The Adam optimization algorithm was chosen for its effectiveness in handling sparse gradients on noisy problems. A notably small learning rate (lr = 0.0001) was implemented to guarantee a stable learning curve throughout the training process. This choice of a lower learning rate helps in fine-tuning the model's adjustments during training, thereby preventing rapid, potentially destabilizing updates to the weights. Furthermore, the GOA played a pivotal role in the optimization of these hyperparameters, significantly contributing to the enhancement of the model's estimation accuracy. The specific values and settings of the hyperparameters mentioned were finalized post-optimization using GOA, ensuring that the model was tuned to its optimal configuration for yield estimation.

#### Comparing models

We compared GCBA with several state-of-the-art models to validate our model's advantage in large-area adaptability yield estimation. These models include SVR, RF, CNN, GRU, and CNN-GRU.

SVR is suitable for complex and nonlinear crop yield estimation. For our study's SVR model, we chose the Radial Basis Function (RBF) kernel, a common approach for handling nonlinear problems. We then found the best values for C (regularization parameter) and gamma (kernel function parameter) through optimization algorithms and fivefold cross-validation. The model was tested on a range of predefined C and gamma values, ultimately selecting values of 100 and 0.1.

RFR is a powerful machine learning model widely used for various estimation tasks, including crop yield estimation, due to its ability to handle high-dimensional features and complex nonlinear relationships. The RFR model in this study also underwent parameter optimization and fivefold cross-validation to find the best parameter combination. The number of trees in the random forest (n_estimators) was set to 200, and the minimum number of samples required at each leaf node (min_samples_leaf) was set to 4.

Traditional CNNs are generally used for image data processing. They extract spatial features through convolutional layers and are suited for data with strong spatial correlations. In yield estimation, CNNs are often used to identify local patterns and relationships in data, especially in time series or spatially dimensional data. For the CNN model structure used in our paper, we employed 2 convolutional layers for feature extraction and a fully connected layer for final estimations.

GRU, a variant of Recurrent Neural Networks (RNN) for processing time series data, addresses the problem of vanishing gradients by introducing update and reset gates, effectively capturing long-term dependencies in time series. In yield estimation, GRU can analyze time-related agricultural data, such as seasonal variations and meteorological conditions. For our comparative GRU model, we used GRU layers combined with a fully connected layer for estimation.

CNN-GRU combines the strengths of CNN and GRU, with CNN extracting local features or patterns in data, and GRU capturing long-term dependencies in time series. For our CNN-GRU model, we added pooling and batch normalization layers after the CNN layer to reduce the model's complexity and overfitting risk and enhance the stability and convergence speed of model training. GRU layers were then used to process time series data. After feature extraction, a fully connected layer was used for final estimations based on these features.

To ensure fairness in comparison and reduce the complexity and resource consumption of experimental design, the three comparative deep learning models (CNN, GRU, CNN-GRU) and our proposed model (GCBA) used the same hyperparameters.

#### Performance evaluation

To rigorously evaluate the performance of all models, we utilized data from the years 2019 and 2020 for testing purposes. This approach was strategically chosen to verify the models' capability to accurately predict yields across different years, particularly focusing on their performance with the most recent data. Such a testing regime is crucial in assessing the generalizability and reliability of the models under varying conditions. For the training of these models, we employed historical data spanning from 2008 to 2018. This extensive timeframe encompasses a wide range of climatic conditions and alterations in the growth environment. Utilizing data over such a prolonged period is instrumental in equipping the models to identify and learn from generalizable patterns. This long-term data exposure ensures that the models are not only attuned to specific year-to-year variations but are also capable of capturing broader, more universal trends relevant to soybean yield estimation.

In terms of model evaluation, we calculated four key metrics: the coefficient of determination (R^2^), the root mean square error (RMSE), the Mean Absolute Error (MAE) and the Mean Absolute Percentage Error (MAPE). These metrics are calculated as follows:

1 $${R}^{2}=1-\frac{\sum_{i=1}^{n}{\left({Y}_{i}-{\widehat{Y}}_{i}\right)}^{2}}{\sum_{i=1}^{n}{\left({Y}_{i}-{\overline{Y}}_{i}\right)}^{2}}$$

2 $$RMSE=\sqrt{\frac{1}{n}\sum_{i=1}^{n}{\left({Y}_{i}-{\widehat{Y}}_{i}\right)}^{2}}$$

3 $$MAE=\frac{1}{n}{\sum }_{i=1}^{n}|{Y}_{i}-{\widehat{Y}}_{i}|$$

4 $$MAPE=\frac{1}{n}{\sum }_{i=1}^{n}\left|\frac{({\widehat{Y}}_{i}-{Y}_{i})}{{Y}_{i}}\right|\times 100\%$$

The coefficient of determination, commonly denoted as R^2^, serves as a key metric for quantifying the correlation between the actual values and those predicted by the model. An R^2^ value approaching 1 indicates a strong predictive ability, signifying that the model effectively captures and explains the variability present in the data. Essentially, a higher R^2^ value reflects the model’s efficiency in fitting the data. Conversely, the RMSE is employed to assess the discrepancies between the model's predicted values and the actual observed values. A lower RMSE value denotes higher predictive accuracy, suggesting that the model's estimations are closely aligned with the actual scenario. The RMSE provides insight into the average magnitude of the estimation errors, thereby serving as a measure of the model’s precision. Additionally, the MAE measures the average magnitude of errors in a set of predictions, without considering their direction. It's a linear score which means all individual differences are weighted equally in the average. The MAPE, on the other hand, provides a perspective on prediction accuracy as a percentage, which can be more intuitive. It's a measure of prediction accuracy of a forecasting method in statistics. It usually expresses accuracy as a percentage. By incorporating these additional metrics, R^2^ and RMSE, alongside MAE and MAPE, the evaluation of the model's performance becomes more nuanced, accounting for both the average error magnitude and the relative error in percentage terms. This comprehensive approach ensures that the model's accuracy and reliability are thoroughly assessed, providing a multifaceted view of its potential for practical application and offering robust decision support for real-world scenarios.

## Results

### Exploratory data analysis

**Figure 3:**
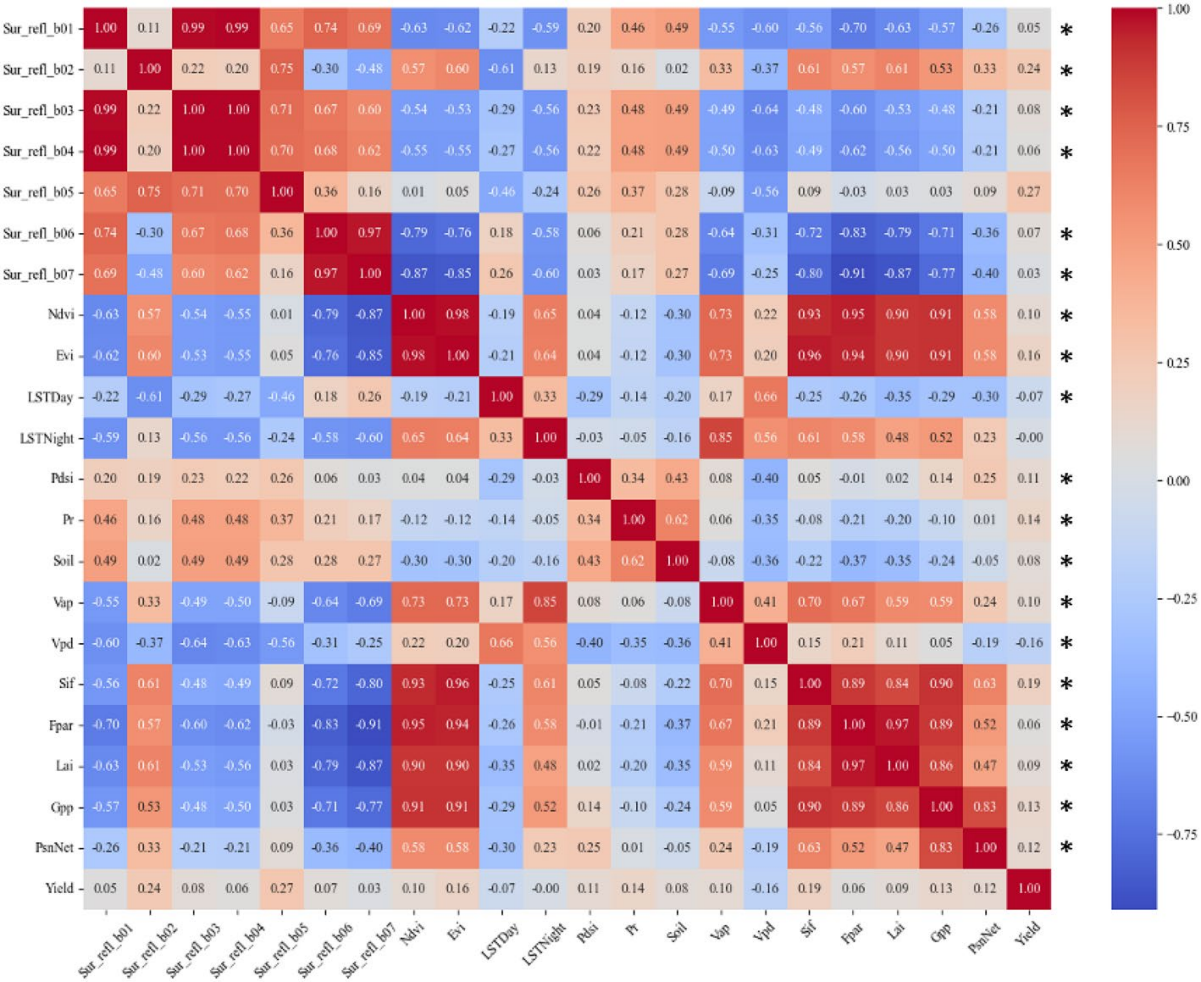
Inter-Variable and Yield Correlation Heatmap with Significance Indicators. The * indicates that the correlation coefficient (r) has a *P*-value less than 0.001, which suggests a very high level of statistical significance for that particular correlation.

In our analysis utilizing crop yield data from 2008 to 2020, we scrutinized the connections between yield and an array of spectral, environmental, and vegetation indices (Fig. 3). This extensive examination revealed a broad spectrum of correlation strengths. Notably, the vast majority of variables, showed P-values less than 0.001, signifying a high level of statistical significance in their relationships with crop yield. Sif, an indicator of plant photosynthetic activity, exhibited a moderate positive correlation (0.1854) with Yield. This reinforces the potential of Sif as a proxy for plant productivity and health. Evi, a more sensitive measure of vegetation health than Ndvi in high biomass regions, had a positive correlation of 0.1570 with Yield, suggesting its utility in capturing crop conditions that are predictive of yield outcomes. Pr showed a positive correlation with Yield (0.1429), which is consistent with the essential role of water availability in crop growth. However, the relatively modest magnitude of this correlation might reflect the complex interplay between precipitation and other environmental factors influencing yield. Gpp and PsnNet, both measures of plant growth and photosynthetic performance, had correlations of 0.1285 and 0.1181, respectively, with Yield. This supports the hypothesis that higher photosynthetic activity is associated with higher yields. Other variables such as Vap, Lai, and Soil moisture content showed positive but weak correlations ranging from 0.0799 to 0.0982. Interestingly, LSTNight had an almost negligible correlation (− 0.0029) with Yield, suggesting that nighttime temperatures in the study region have minimal linear impact on yield. Conversely, LSTDay exhibited a weak negative correlation (-0.0678) with Yield, hinting that higher daytime temperatures might be linked to lower yields, possibly due to heat stress. Despite their varying degrees of correlation, both LSTNight and LSTDay are retained in the analysis as they can reflect the impact of extreme weather conditions on yield to a certain extent. Lastly, Vpd showed a moderate negative correlation (-0.1642) with Yield, which is significant as Vpd is a measure of atmospheric moisture stress that can affect plant transpiration and overall health.

### Comparison of models for county-level yield estimation

In our study, we conducted a comparative analysis of six distinct estimation models—SVR, RFR, CNN, GRU, CNN-GRU, and GCBA—focusing on their ability to predict county-level soybean yields in the United States for the years 2019 and 2020. The performance evaluation, detailed in (Table 2), reveals that the GCBA model outperformed the other models in both test years. The superior performance of the GCBA model is particularly pronounced across all four performance metrics—R^2^, RMSE, MAE, and MAPE. In 2019, the GCBA model not only achieved an RMSE of 4.3288 bushels per acre and an R^2^ value of 0.6873, but it also demonstrated impressive results in terms of MAE and MAPE. The model recorded an MAE of 3.2712 bushels per acre and a MAPE of 6.88%, further emphasizing its predictive accuracy in estimating soybean yields. In the subsequent year, 2020, the GCBA model maintained its superior performance, yielding an RMSE of 4.4612 bushels per acre and an R^2^ of 0.7057. Its consistency was also reflected in the MAE and MAPE metrics, with the model achieving an MAE of 2.8684 bushels per acre and a MAPE of 5.80%. These results underscore the significant predictive accuracy of the GCBA model, demonstrating its robustness and reliability in soybean yield estimation.

**Table 2:** Soybean yield estimation performance of the GCBA and comparing models.

Year	Model	Training dataset				Testing dataset			
		R^2^	RMSE (bushels/acre)	MAE (bushels/acre)	MAPE (%)	R^2^	RMSE (bushels/acre)	MAE (bushels/acre)	MAPE (%)
2019	SVR	0.6672	5.3363	3.9256	8.79	0.4675	5.6489	4.1651	8.48
	RFR	0.8844	3.1936	2.2666	5.26	0.5320	5.2956	4.0861	8.71
	CNN	0.8906	3.0602	2.0637	4.53	0.5840	4.9927	3.7847	7.89
	GRU	0.9035	2.8733	1.9392	4.25	0.6391	4.6507	3.5475	7.31
	CNN-GRU	0.9114	2.7541	1.8465	4.04	0.6633	4.4919	3.4383	7.27
	GCBA	0.9203	2.6121	1.7547	3.83	0.6873	4.3288	3.2712	6.88
2020	SVR	0.6743	5.2793	3.8565	8.63	0.4082	6.3444	4.1689	8.46
	RFR	0.8295	3.8202	2.6299	5.77	0.5364	5.5906	3.7262	7.65
	CNN	0.8143	3.9865	2.7777	6.11	0.6152	5.0943	3.1645	6.32
	GRU	0.8616	3.4415	2.3305	5.10	0.6567	4.8264	3.0281	6.08
	CNN-GRU	0.9032	2.8788	1.9162	4.16	0.6671	4.7926	2.9962	5.93
	GCBA	0.9255	2.5251	1.6805	3.62	0.7057	4.4612	2.8684	5.80

In contrast to the GCBA model, the other models tested in our study, including SVR, RFR, CNN, GRU, and CNN-GRU, exhibited relatively lower performance levels in both 2019 and 2020. For instance, the SVR model recorded an RMSE of 5.6489, an R^2^ of 0.4675, MAE of 4.1651, and MAPE of 8.48 in 2019, which further declined in 2020 to an RMSE of 6.3444, an R^2^ of 0.4082, MAE of 4.1689, and MAPE of 8.46. Similarly, the RFR, CNN, and GRU models exhibited variations in their performance across these metrics, yet none reached the predictive capability demonstrated by the GCBA model. The CNN-GRU model, which integrates the strengths of both CNN and GRU, showed relatively better performance, with an RMSE of 4.4919, an R^2^ of 0.6633, an MAE of 3.4383, and a MAPE of 7.27 in 2019, and demonstrated further improvement in 2020 with an RMSE of 4.7926, an R^2^ of 0.6671, an MAE of 2.9962, and a MAPE of 5.93. This improvement underscores the model's efficacy in processing time series data. Despite this enhanced performance, the CNN-GRU model still fell short of the GCBA model's superior predictive accuracy. Overall, while the combined CNN-GRU model demonstrated an improvement over the individual CNN or GRU models, it is the GCBA model that consistently led in terms of overall estimation accuracy for soybean yields in both test years.

**Figure 4:**
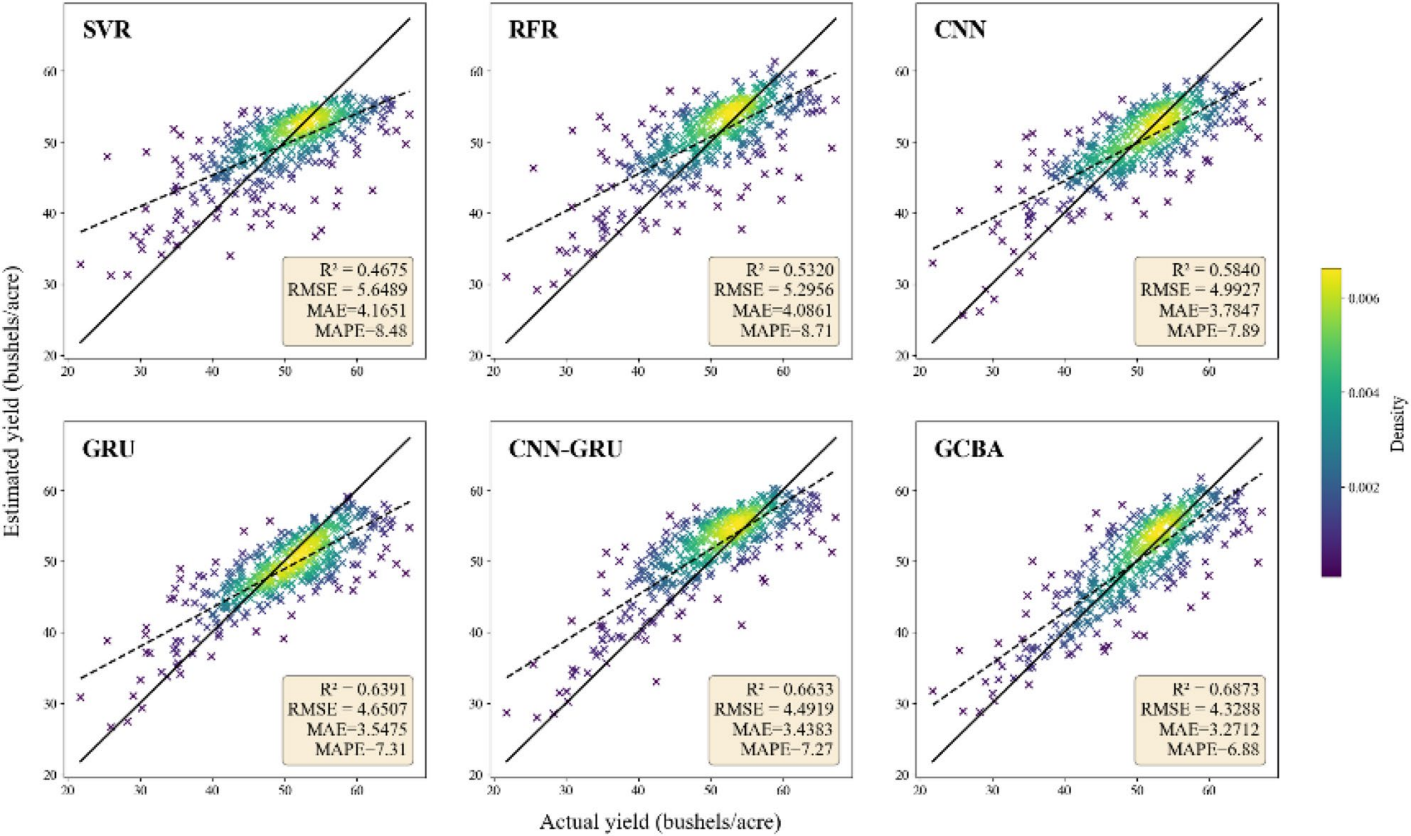
Scatter plot of estimated yield versus actual yield for the year 2019. The solid line represents the diagonal of y = x, indicating that the estimated production is equal to the actual production. The dashed line is the fitted trend line. The color indicates the density of points, where purple represents low point density and yellow indicates high point density.

**Figure 5:**
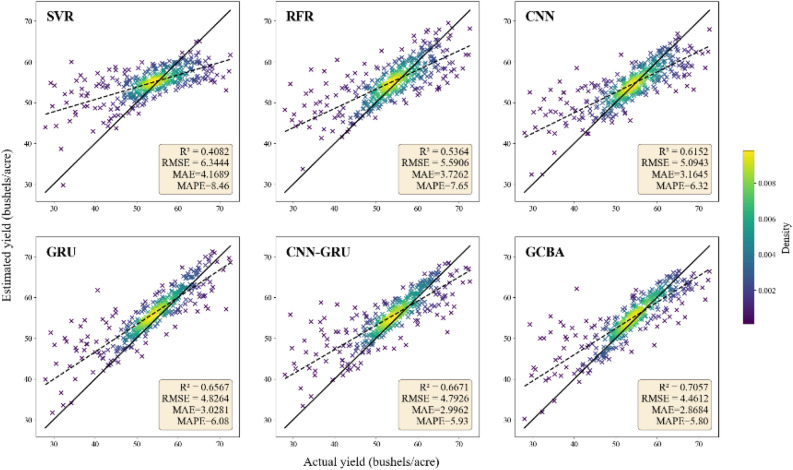
Scatter plot of estimated yield versus actual yield for the year 2020.

Figures 4 and 5 effectively visualize the correlation between the actual soybean yields and the yields predicted by the various models. In these figures, the proximity of the data points to the y = x diagonal line serves as an indicator of estimation accuracy. A closer alignment of the points to this line denotes higher accuracy in the yield estimations. Observing the density plots in these figures, it becomes apparent that a majority of the predicted values from the models are clustered near the diagonal line. This clustering is particularly pronounced for the GCBA model. In both years' data, the GCBA model exhibits the most concentrated aggregation of points near the diagonal line. This visual representation aligns with the quantitative findings reported in the table, where the GCBA model exhibits superior performance across all four metrics: R^2^, RMSE, MAE, and MAPE. The tight clustering of points for the GCBA model in the plots visually reinforces its exceptional accuracy in predicting soybean yields, as quantitatively evidenced by its leading R2 and RMSE values.

These results validate the GCBA model's strong potential in large-scale crop yield estimations and highlight its capability to balance overestimations and underestimations effectively. The model's consistent performance across different years demonstrates the effectiveness of integrating deep learning and optimization algorithms in agricultural yield estimation, underlining the importance of this approach in enhancing predictive accuracy and advancing the field.

### Spatial patterns of county-level yield estimation

**Figure 6:**
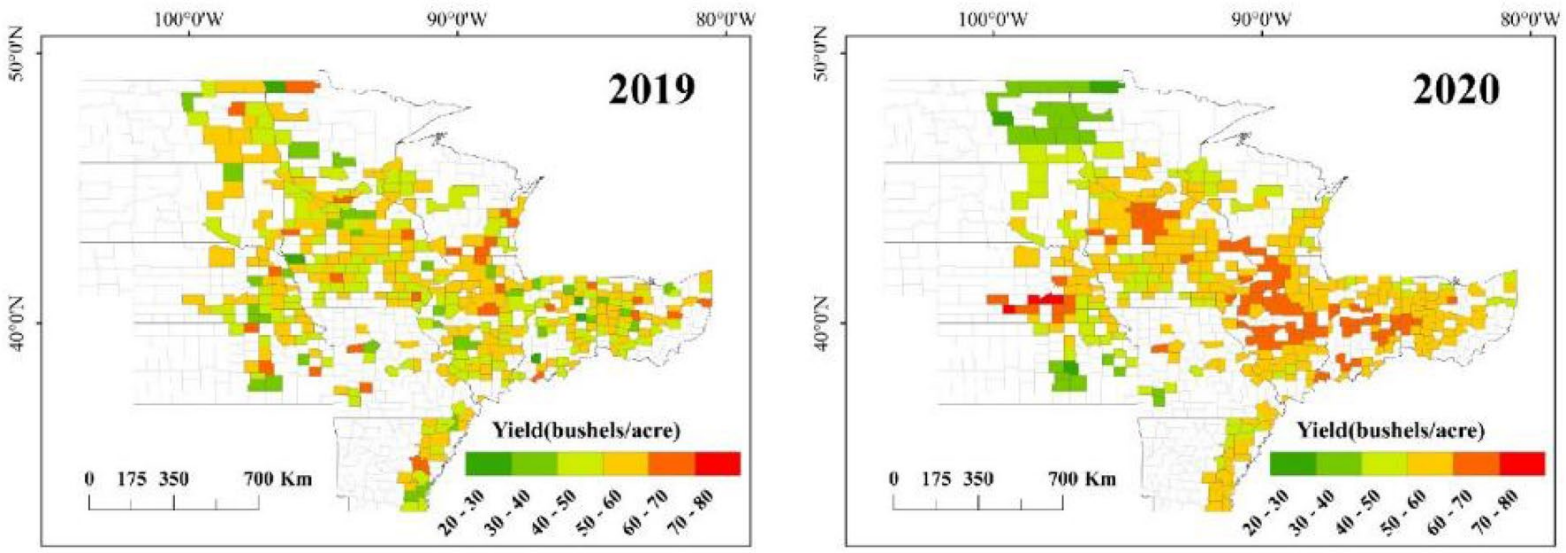
Actual yield maps for 2019 and 2020.

**Figure 7:**
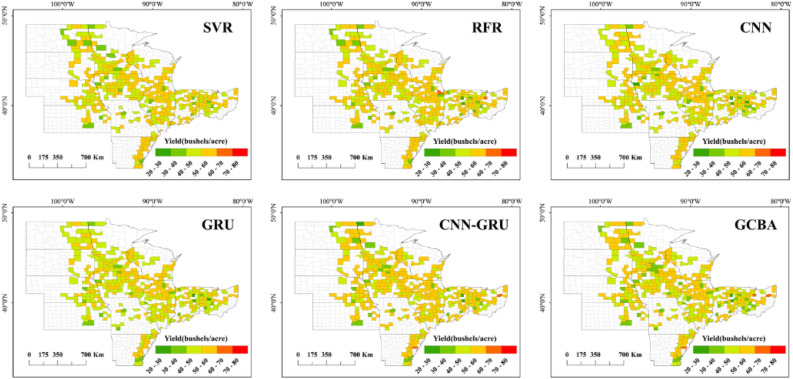
Spatial pattern of yield estimations for 2019 by SVR, RFR, CNN, GRU, CNN-GRU and GCBA.

**Figure 8:**
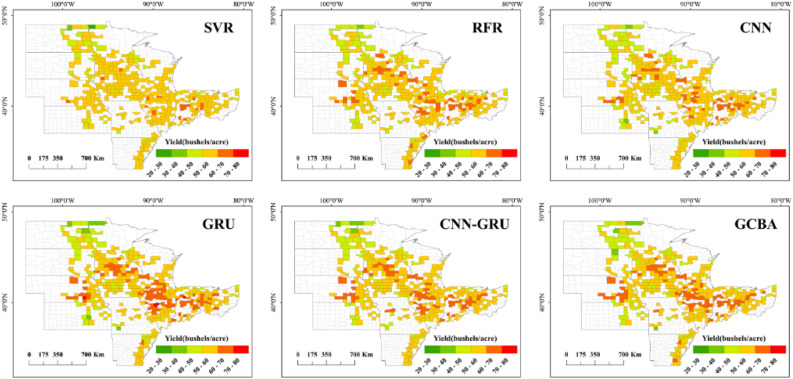
Spatial pattern of yield estimations for 2020 by six models.

As depicted in Fig. 6 our examination of the official soybean yields for 2019 and 2020 offers a fundamental insight into the spatial distribution of crop productivity across the study area. Notably, counties with high soybean yields are predominantly clustered in the central region, a trend that has remained consistent over the past two years. Conversely, counties with lower yields are generally more scattered, mostly situated on the periphery of the study area. Figures 7 and 8 further delineate the spatial patterns of soybean yields as predicted by six different models for the years 2019 and 2020. Among these, the CNN-GRU and GCBA models were observed to have higher accuracy in predicting soybean yields for both years. The GCBA model, in particular, demonstrated exceptional proficiency in predicting the distribution of high-yield counties within the central region. Its estimations closely aligned with the actual official county-level soybean yields, thereby showcasing its superior spatial estimation capabilities. In comparison to traditional models such as SVR and RFR, the GCBA model exhibits greater efficiency in processing and analyzing multi-source remote sensing data. This enhanced capability is reflective of its advanced algorithmic composition, making it a more effective tool for predictive analysis in agricultural contexts.

**Figure 9:**
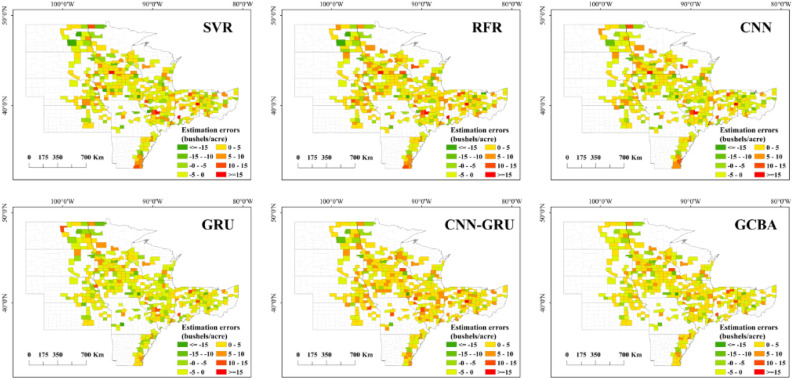
County-level yield estimation error maps for 2019 by SVR, RFR, CNN, GRU, CNN-GRU and GCBA.

**Figure 10:**
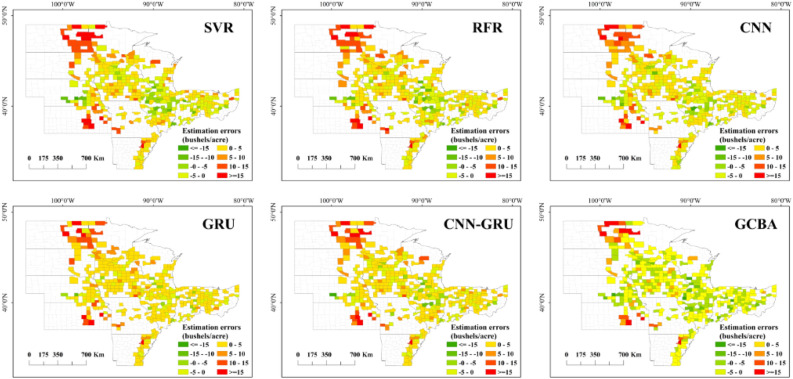
County-level yield estimation error maps for 2020 by six models.

The analysis of the error maps for 2019, as illustrated in Fig. 9, reveals a certain level of uniformity among the models, with a general tendency to underestimate soybean yields. Notably, the GCBA model distinguishes itself at lower error thresholds, showcasing its heightened accuracy in estimations. This superior performance can be attributed to the model’s adeptness in integrating complex interactions within multi-source remote sensing data. In the 2020 analysis, depicted in Fig. 10, the error distribution across all models appears broadly similar, particularly with a common underestimation trend in the central region. However, an observable deviation is seen in the northern counties, where there is a significant overestimation of yields by all models. The distribution and error maps of soybean yield estimations for both 2019 and 2020 demonstrate that the GCBA model maintains high predictive accuracy in each year. In contrast, traditional machine learning methods such as SVR and RFR exhibit less optimal performance. These findings not only underscore the GCBA model’s proficiency in predicting spatial distribution but also highlight its superiority in effectively processing and assimilating multi-source remote sensing data, as well as in capturing various factors that influence crop growth. The results of our study showcase the GCBA model’s potential for practical applications, particularly in regional crop yield estimation and agricultural resource management.

### Performance of variables in county-level soybean yield estimation

#### Yield estimation using different variable combinations

**Figure 11:**
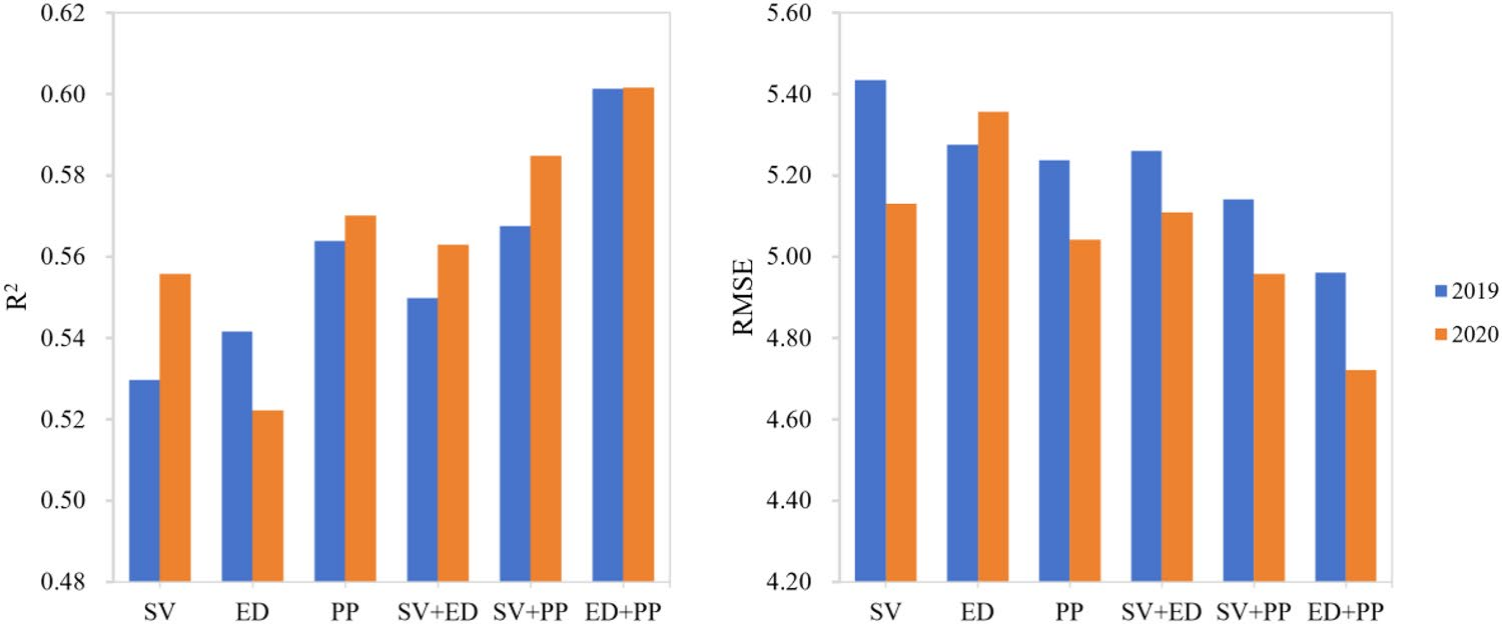
Performance of various data combinations in predicting county-level soybean yield using the GCBA model.

In our research, we employed the high-performing GCBA model for estimating soybean yields. This model's application involved the use of multiple indicators, including surface reflectance and vegetation indices (SV), environmental data (ED), and photosynthesis-related parameters (PP). We assessed the impact of these variables, both individually and in various combinations, on the accuracy of yield estimation. This assessment was quantified using two key metrics: the R^2^ and the RMSE. The resulting data and insights are presented in Fig. 11.

In the year 2019, the R^2^ for the SV variable was recorded at 0.5297, showing a slight improvement to 0.5558 in 2020. For the ED variable, R^2^ values were 0.5417 and 0.5223 in 2019 and 2020, respectively, indicating a relatively weaker performance compared to SV. The PP demonstrated a consistent R^2^ of around 0.57 in 2019, underscoring their substantial explanatory power in yield estimation when used independently. Upon combining these variables, we observed an enhanced predictive performance. The SV + ED combination yielded an R^2^ of 0.5498 in 2019, increasing to 0.5629 in 2020, suggesting that the integration of variables can improve model accuracy. More notably, the SV + PP combination showed higher R^2^ values in both years, at 0.5675 and 0.5848, respectively. The combination of ED + PP demonstrated the most remarkable performance, surpassing an R^2^ of 0.60 in both 2019 and 2020, marking it as the most effective variable combination among those tested.

Regarding the RMSE, the SV variable recorded a value of 5.4343 bushels/acre in 2019, which showed an improvement, decreasing to 5.1307 bushels/acre in 2020. For the ED variable, the RMSE experienced slight fluctuations but predominantly hovered between 5.2 to 5.4 bushels/acre across the two years. Meanwhile, the PP variable demonstrated an RMSE of 5.2378 bushels/acre in 2019, which further improved to 5.0417 bushels/acre in 2020. This indicates that photosynthesis-related parameters alone can achieve relatively high estimation accuracy. When examining the combined variables, a similar trend in RMSE was observed. The SV + ED combination registered an RMSE of approximately 5.3 bushels/acre in 2019, which then reduced to around 5.1 bushels/acre in 2020. The RMSE values for the SV + PP and ED + PP combinations in 2019 were 5.1415 and 4.9607 bushels/acre, respectively, showing a decrease in 2020 to 4.9576 and 4.7217 bushels/acre. These results demonstrate the effectiveness of combining variables in reducing estimation errors, with the combined use of variables leading to a consistent decrease in RMSE values over the years, thereby enhancing the predictive accuracy of the model.

#### Importance of Individual Indicators in yield estimation

**Figure 12:**
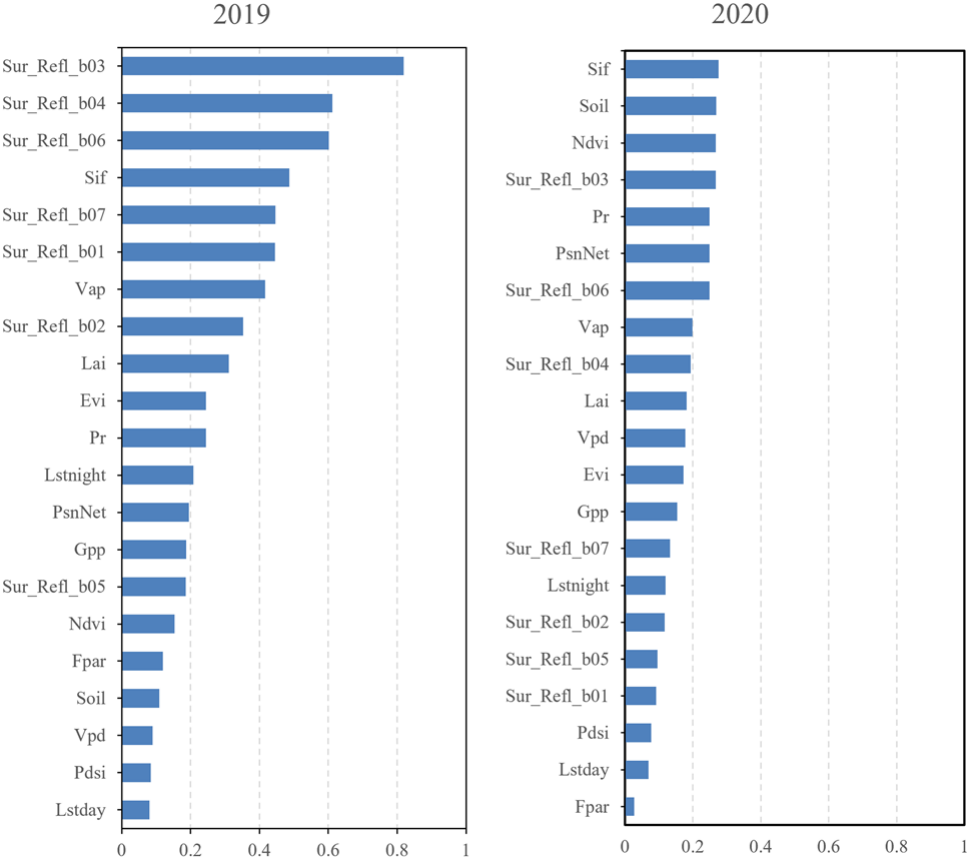
Importance of individual indicators obtained through SHAP analysis of the GCBA model.

In our soybean yield estimation models for 2019 and 2020, the significance of individual variables was quantitatively assessed through their feature importance scores. These scores were derived using the GCBA model in conjunction with SHAP (SHapley Additive exPlanations). These scores provide a measure of each variable's relative contribution and significance in the predictive model (Fig. 12).

For the 2019 model, the surface reflectance band Sur_refl_b03 emerged as the most influential variable, registering the highest importance score of 0.8185. This was followed by Sur_refl_b04 and Sur_refl_b06, with importance scores of 0.6124 and 0.6020, respectively. These findings underscore the critical role of surface reflectance features in soybean yield estimation. Additionally, the photosynthesis-related variable Sif demonstrated its substantial influence with an importance score of 0.4869. In the 2020 model, the Sif variable's importance score increased to 0.2756, marking it as one of the primary factors influencing the estimation model. Concurrently, the Soil variable recorded an importance score of 0.2695, highlighting the significant impact of soil conditions on the model's estimations. Other variables such as NDVI and Pr also played pivotal roles, with scores of 0.2679 and 0.2493, respectively. The variation in the distribution of feature importance between 2019 and 2020 suggests the influence of inter-annual climatic changes on soybean yield, reflecting the dynamic nature of agricultural ecosystems and the necessity to adapt predictive models accordingly.

## Discussion

In this research, we developed and validated a novel CNN-BiGRU deep learning model, augmented by the optimization capabilities of the GOA and the precision of an attention mechanism. This model's success in integrating multi-source remote sensing data enabled us to accurately estimate soybean yields at the county level across the United States. The adoption of this methodology not only improved yield estimation accuracy but also offered fresh insights into complex agricultural data analysis. Our model was trained using data spanning from 2008 to 2018, and its performance was evaluated based on soybean yield estimations for 2019 and 2020. The GCBA model showcased distinct advantages. Firstly, the integration of GOA for parameter optimization significantly enhanced the model’s training efficiency and convergence, aligning with Mirjalili et al.’s findings on GOA's efficacy^57^. The CNN component of our model demonstrated its proficiency in handling multi-dimensional data, a capability that is widely acknowledged in the literature^58,59^. Furthermore, the BiGRU structure’s ability to capture long-term dependencies in time series data provided an in-depth understanding of crop yield dynamics over time^60^. The incorporation of the attention mechanism in our model was particularly effective when applied to multi-source data analysis. It automatically emphasized features most relevant to soybean yield estimation, vital for processing extensive remote sensing datasets. This not only boosted estimation accuracy but also improved the interpretability of influential factors^61,62^. Practical application results revealed that the GCBA model outperformed both traditional machine learning and other deep learning models in terms of accuracy and reliability in predicting soybean yields for 2019 and 2020 (Figs. 4, 5).

Given the extensive nature of the study area and the diverse climatic conditions across regions, this research strategically selected data from the months of May to August for the years 2008 to 2020. This timeframe is critical as it encompasses the key stages of the soybean growing season, including the crucial flowering and pod-setting phases. During this period, the information gleaned from remote sensing data, such as surface reflectance, vegetation indices, environmental data, and photosynthesis-related parameters, plays a significant role in influencing soybean growth conditions and ultimately, their final yield. Reflectance and vegetation indices are pivotal in assessing plant health and growth dynamics. They provide insights into crop biomass and photosynthetic efficiency^63^. Additionally, environmental data, including LST and Pr, alongside photosynthesis parameters like Sif and Fpar, are intricately connected to the crop's growth conditions and physiological status^64,65^. By comprehensively analyzing these remote sensing and environmental parameters, our study adeptly captures the essential factors that influence soybean yield. This integrative approach substantially improves the accuracy and reliability of early yield estimations. The analysis of data from this vital growth period enables more effective estimations of the entire growing season's yield. It takes into account regional climatic variations and environmental disparities, thus enhancing the precision and practical applicability of our estimations. Hence, this study not only concentrates on determining the final yield of soybeans but also delves into the potential methodologies for early yield forecasting, highlighting the significance of timely and informed agricultural decision-making.

To elucidate the specific contributions of each parameter within the GCBA model for soybean yield estimation, we employed SHAP value analysis. This approach not only enhanced our comprehension of the model’s predictive capabilities but also illuminated the connections between remote sensing indicators and soybean yield. In the year 2019, SHAP analysis identified the surface reflectance band 3 (Sur_Refl_b03) as having the highest feature importance score, closely followed by bands 4 (Sur_Refl_b04) and 6 (Sur_Refl_b06). This result underscores the pivotal role of specific surface reflectance bands in the remote sensing data for yield estimation. Additionally, Sif also emerged as a variable of high importance, highlighting the influence of vegetation growth conditions and biomass accumulation on yield estimation. Conversely, in 2020, Sif ascended to the top of the importance ranking, marginally surpassing Soil and Ndvi. This shift suggests that in 2020, variables associated with vegetation activity and soil characteristics exerted a more pronounced impact on yield estimation. While Sur_Refl_b03 continued to be significant, its relative importance decreased compared to 2019. The variation in key variables across different years reflects environmental changes and their impact on crop yield estimation. The analysis for 2019 highlighted the criticality of remote sensing band data in monitoring crop biomass and growth conditions. However, in 2020, the onset of extreme climate events shifted the focus to photosynthesis parameters and soil characteristics as predominant influencing factors. This change aligns with the United States experiencing high temperatures and drought conditions. These environmental stressors accelerated crop maturation and limited water availability, thereby affecting photosynthetic efficiency and growth. In light of these extreme climate conditions, the importance of individual indicators for yield estimation was relatively lower in 2020. This observation suggests that under such conditions, no single environmental or biological factor can singularly predict crop yield accurately. It reflects the complexity and multifactorial nature of agricultural systems, particularly under varying climate conditions.

**Figure 13:**
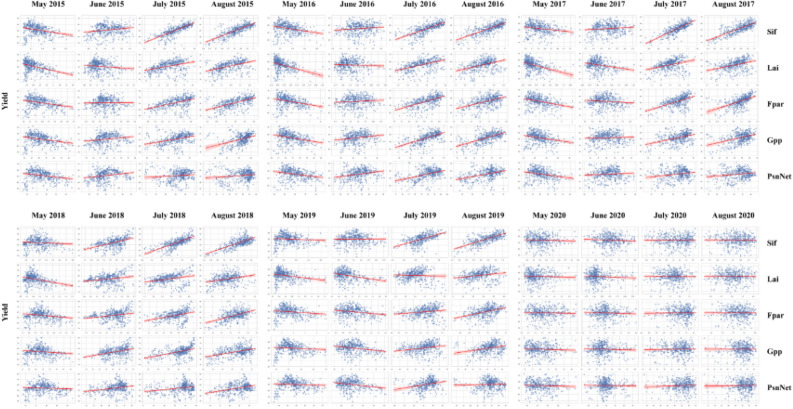
Relationship between photosynthesis-related parameters and actual yield from 2015 to 2020.

In our study, the incorporation of PP in the GCBA model, both as standalone factors and in conjunction with SV, as well as ED, showed substantial predictive power. This finding underscores the importance of the various photosynthesis-related parameters utilized in this study—Sif, Lai, Fpar, Gpp, and PsnNet—in soybean yield estimation. To further comprehend how these photosynthetic parameters influence crop yield, we undertook a detailed analysis comparing these parameters against actual yield data from May to August of 2015 to 2020. Our analysis revealed a consistent pattern across most years: as the growing season progressed, the photosynthesis-related parameters gradually increased, correlating more strongly with yield. This trend is visually represented in scatter plots (Fig. 13), where an upward slope is observable. Additionally, a corresponding increase in the correlation coefficients of these parameters with yield is noted in data tables (Table 3). By August, this correlation typically reached its peak. This observed pattern is in line with the general plant growth cycle. During the peak growth and reproductive phases, photosynthetic activity intensifies, leading to increased biomass accumulation and potential yield enhancement. Such findings offer valuable insights into the dynamics of crop growth and productivity, enhancing our ability to accurately predict crop yields based on photosynthetic parameters.

**Table 3:** Correlation coefficients between photosynthesis parameters and actual yield data from May to August, 2015 to 2020.

	May 2015	June 2015	July 2015	August 2015	May 2016	June 2016	July 2016	August 2016	May 2017	June 2017	July 2017	August 2017
Sif	0.2739	0.1943	0.7722	0.7829	0.2826	0.1254	0.7084	0.7473	0.2001	0.1166	0.7947	0.7850
Lai	0.3721	0.1220	0.3796	0.4543	0.3669	0.0516	0.4771	0.4198	0.3860	0.2345	0.3365	0.3797
Fpar	0.2850	0.0116	0.4851	0.5739	0.2983	0.0836	0.5860	0.4874	0.3340	0.1025	0.5130	0.5409
Gpp	0.2618	0.1794	0.3749	0.4186	0.2938	0.1844	0.5934	0.5961	0.2484	0.0453	0.4000	0.4163
PsnNet	0.2269	0.2160	0.1145	0.1490	0.2455	0.2399	0.4207	0.3968	0.2298	0.0786	0.2126	0.1836

	May 2018	June 2018	July 2018	August 2018	May 2019	June 2019	July 2019	August 2019	May 2020	June 2020	July 2020	August 2020
Sif	0.0788	0.4823	0.7287	0.7005	0.0841	0.0088	0.5522	0.7126	0.0438	0.0720	0.0119	0.0151
Lai	0.3098	0.2163	0.3034	0.2857	0.2355	0.3102	0.0483	0.2236	0.0334	0.0725	0.0080	0.0056
Fpar	0.1876	0.2428	0.3568	0.4919	0.1456	0.2066	0.1692	0.4205	0.0373	0.0606	0.0199	0.0014
Gpp	0.1298	0.3586	0.4499	0.4332	0.0914	0.2321	0.2419	0.2835	0.0540	0.0467	0.0285	0.0008
PsnNet	0.0762	0.2416	0.2581	0.3467	0.0601	0.2302	0.2798	0.0259	0.0537	0.0153	0.0599	0.0196

However, data from 2020 showed a deviation from this pattern, likely reflecting the impact of special factors on photosynthetic parameters or yield. That year, several major agricultural regions in the U.S. experienced extreme weather events, including severe droughts and the Derecho storm in August, which had widespread impacts on crops^66–68^. The drought significantly affected photosynthetic parameters like Sif, Lai, Fpar, GPP, and PsnNet, as they are closely related to plant health and photosynthetic capacity. The lack of water not only reduced leaf area but also hindered the plant's ability to perform photosynthesis, thereby decreasing crop yield. The Derecho storm, with its strong winds and potential hail, caused physical damage to plants, reducing effective leaf area and thus affecting GPP and PsnNet. Additionally, high temperatures stressed plants, reducing photosynthetic efficiency, and heat stress might have accelerated plant development, leading to premature maturation and reducing the time for biomass accumulation^69–71^. Thus, the impact of photosynthetic parameters on yield was less in 2020, possibly indicating that extreme weather conditions inhibited normal photosynthetic processes and growth, weakening the usual correlation between parameters and yield^72^. The stress caused by drought and high temperatures might have put plants in a non-ideal photosynthetic state, whereas typically, these parameters are closely linked to yield under normal growth conditions. Scatter plots clearly show the adverse effects of extreme climatic events on plant growth conditions during the 2020 growing season, potentially weakening the expected positive correlation between photosynthetic activity and crop yield.

The findings from our analysis lead to the conclusion that photosynthesis-related parameters can be effectively utilized to predict crop yield to a significant degree. In typical years, as the growing season progresses, there is a discernible positive correlation between the accumulation of photosynthetic parameters and an increase in crop yield. This observation implies that by closely monitoring the photosynthetic parameters of plants, it is feasible to estimate the potential yield of crops. Such estimates are invaluable for providing guidance in agricultural production and facilitating precision agricultural management. However, it is crucial to acknowledge that under extreme climatic conditions, this predictive relationship may become less reliable^73,74^. The 2020 scenario serves as a pertinent example, where extreme weather events altered the usual relationship between photosynthetic parameters and yield. This change led to a reduction in the accuracy of yield estimations based on these parameters. Consequently, it becomes imperative to factor in environmental influences and climate variability that affect crop growth when employing photosynthetic parameters for yield estimation. Recognizing and accounting for these factors is essential to ensure the accuracy and reliability of yield forecasts, particularly in the face of increasing climatic extremes.

In our study, we carried out a detailed analysis of the spatial distribution of yield estimation errors to discern regional disparities in the predictive performance of the models. Upon examining the error maps for county-level yield estimates, it was observed that most models tend to underestimate yields in certain regions. This underestimation could be attributed to the models' limited ability to fully encapsulate critical factors influencing yields in these areas, such as specific climatic conditions, soil types, planting practices, and crop varietals. A comparison between the official yield map and the error map for 2020 revealed that yields in northern counties were generally lower, yet the estimation models exhibited a trend of overestimation in these areas. This discrepancy is likely linked to the severe drought conditions experienced in 2020 across the northern plains of the United States, particularly in states like ND, SD, MN^75^. Soybeans, being sensitive to water availability, crucially depend on moisture during key growth stages like flowering and pod-setting. The drought conditions in these regions resulted in inadequate soil moisture, inducing water stress and adversely impacting the normal growth of soybeans. This stress led to slower plant development, flower and pod shedding, and consequently, a significant reduction in yields^76,77^. Additionally, drought conditions can adversely affect seed development and quality, and heighten the plants' vulnerability to diseases and pests^78,79^. Consequently, the models may have overestimated yields by not sufficiently accounting for the unique extreme drought conditions and related disease and pest issues prevalent in these regions. Looking ahead, integrating climate models and real-time environmental monitoring data could substantially improve the accuracy of crop yield estimations. Such enhancements are crucial for effectively addressing the challenges posed by climate change and its impact on agriculture.

## Conclusions

In this research, we developed an advanced deep learning framework, termed GCBA, to predict soybean yields at the county level across the United States. This framework, which incorporates a CNN-BiGRU-Attention model optimized by the GOA, was applied to multi-variable remote sensing data. We benchmarked the GCBA model against five other machine learning and deep learning models to assess its efficacy in yield estimation over large areas and extended time series. Our results indicate that the GCBA model exhibits superior performance in comparison to the other models. Specifically, in the tests conducted for the years 2019 and 2020, the GCBA model demonstrated superior performance over its counterparts, excelling in all four key metrics: RMSE, R^2^, MAE, and MAPE. For the year 2019, the GCBA model achieved an RMSE of 4.3288 bushels/acre, an R^2^ of 0.6873, an MAE of 3.2712 bushels/acre, and a MAPE of 6.88%. In 2020, it continued its impressive performance with an RMSE of 4.4612 bushels/acre, an R^2^ of 0.7057, an MAE of 2.8684 bushels/acre, and a MAPE of 5.80%. These results underscore the GCBA model's notable accuracy in yield estimation. Moreover, our study demonstrates that the integration of diverse data sources significantly enhances the precision of yield estimation models. Among these, photosynthesis-related parameters emerged as pivotal for accurately predicting soybean yield. Monitoring indicators of plant photosynthetic activity enables more precise estimates of potential crop yields, providing a robust scientific foundation for agricultural production decision-making and the implementation of precise agricultural management practices.
